# Supplementary material for: Effects of Microbeam Irradiation on Rodent Esophageal Smooth Muscle Contraction
Source: Cells. 2022 Dec 31;12(1):176. doi: 10.3390/cells12010176 (PMC9818134; doi:10.3390/cells12010176)
Supplement: Supplementary file 1 [file cells-12-00176-s001.zip › cells-2069453-supplementary.pdf]

Appendix A (Supplementary data)

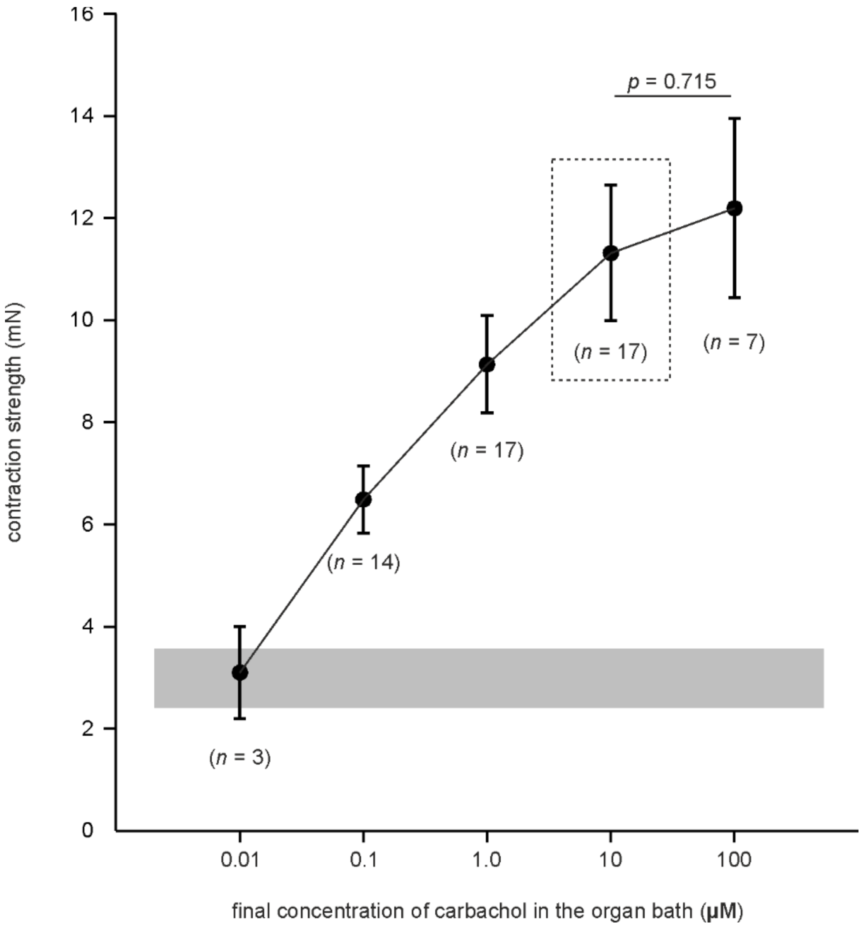

Figure S1: Effects of carbachol on esophageal smooth muscle contraction force. The baseline tone was not statistical different among the segments. With 0.01 μM CCH, no contraction could be induced, and the contraction strength corresponded to the baseline tone (gray box). With increasing concentrations, the contraction strength amplified and became maximal with a CCH concentration of 10 μM and 100 μM. There was no statistic difference between these two concentrations ($p = 0.715$, student's *t*-test). The baseline tone of all segments is highlighted by the grey box, and the dashed rectangle highlights the carbachol concentration we used in our experiments. *p*-values were calculated with a student's *t*-test resp. with a one-way ANOVA test. The number of segments we used to test the different concentrations is given in parentheses. CCH = carbachol.
